# Supplementary material for: Identification of Intervention Opportunities through Assessment of the Appropriateness of Antibiotic Prescribing in Surgical Patients in a UK Hospital Using a National Audit Tool: A Single Centre Retrospective Audit
Source: Antibiotics (Basel). 2022 Nov 8;11(11):1575. doi: 10.3390/antibiotics11111575 (PMC9686826; doi:10.3390/antibiotics11111575)
Supplement: Supplementary file 1 [file antibiotics-11-01575-s001.zip › File S2-Antibiotic Appropriateness Survey Instrument V9.pdf]

ANTIBIOTIC APPROPRIATENESS – END OF COURSE ASSESSMENT – DRAFT v8.1_np

Survey to be performed for a single episode of infection in hospitalised ADULTS at the end of a course of therapy (or if stop-date confirmed)

IMPORTANT: data fields are optional and designed to support decision-making overleaf

DEMOGRAPHICS		
Survey number:	Hospital number:	
Survey date:	Admission date:	Discharge date:
Your name:	Age:	
Your profession:	Gender:	
Infection specialist:	Ward:	
Data collection time: mins	Hospital specialty:	

MEDICAL CASE NOTES
Presenting complaint:
History of presenting complaint:
Relevant past medical history:
Immunocompromised? †
Relevant family or social history:
Relevant drug history:
Allergy to antibiotics:
Urine dipstick test result:
Evidence of infection at anatomical site?
Details of evidence of infection from systems review and examination:
**WORKING infection diagnosis:**
Progress notes over next 2-3 days:
Review of antibiotic prescription within 72h?
Prescribing decision at pre-72h review:
**FINALISED infection diagnosis:**
Local standard course length for infection: days
Explanation for prolonged antibiotic course?

LABORATORY RESULTS (include date)			
Record most significant laboratory results **before starting antibiotics** & during therapy	Pre-start	Day 2-3	Day 5-7
C-Reactive Protein (CRP):			
White Blood Cell (WBC) count:			
Lactate:			
Other relevant laboratory results:			

MICROBIOLOGY	
Blood cultures: Y / N	Pathogen identified: Y / N
Other specimen cultures: Y / N	Pathogen identified: Y / N
Has there been a discussion with Medical Microbiology?	
Details (include date, specimen type, pathogen ID, susceptibilities):	

MEDICAL IMAGING
Details (include date, imaging modality, findings):

**Vital Signs & National Early Warning Score (NEWS-2)**			
**Physiological Parameters**	**Pre-start**	**Day 3**	**Day 5-7**
Respiration Rate (per minute)			
SpO_2 (%)			
Air or oxygen?			
BP (mmHg)			
Pulse (per minute)			
Consciousness (ACVPU)			
Temperature (°C)			
**NEWS-2 Score:**			

†**Definition of immunocompromised** consistent with the "Green Book". i.e. Any of: Immunodeficiency syndrome; HIV infection; Bone marrow or stem cell transplant; Chemo / radiotherapy within 6 months; High-dose steroids >/=40mg prednisolone/day for >7days; or Immunosuppressant drugs.

**Infection specialist judgment of appropriateness of antibiotic prescribing**		If "No", document explanation and estimate excess days of therapy in the table below

**A. Was antibiotic treatment indicated from the outset?**<br>i.e. Was it reasonable to start antibiotic treatment under the circumstances?<br>• Consider only the information available to the prescriber at the time of prescribing (e.g. symptoms/signs; vital signs; laboratory results; urine dipstick; near-patient tests; microbiology; imaging)<br>• Do not take antibiotic spectrum into consideration; judge only whether any antibiotic was indicated	Y	Comments:	
**B. Was antibiotic treatment indicated beyond the post-prescription (48-72h) review?**<br>i.e. Was it reasonable to continue antibiotics beyond 72 hours?<br>• Consider only the information available to the prescriber at the time of the post-prescription review (e.g. symptoms/signs; vital signs; laboratory results; microbiology; imaging)	N		
**C. Was antibiotic treatment indicated beyond the standard treatment duration for the infection?**<br>i.e. Was it reasonable to continue antibiotics beyond the standard course length in local guidelines?<br>• Document any explanation for prolonged treatment (e.g. persistent symptoms, uncontrolled source)	n		

**DRUG CHART / PRESCRIPTION**						**Record any non-essential (excess) days of therapy (DOTs) for one of 3 reasons**		
**Antibiotic**	**Drug name (route & dose regimen)**	**Start date* & time**	**Stop date* & time**	**Days of therapy (DOTs)¥**	**Comments**	A. Antibiotic(s) not indicated at outset	B. Unexplained continuation beyond 72 hours	C. Unexplained continuation beyond standard duration
**1**				0 days		0 days	days	days
**2**				0 days		0 days	days	days
**3**				0 days		0 days	0 days	0 days
**4**				0 days		0 days	0 days	0 days
**5**				0 days		0 days	0 days	0 days
**6**				0 days		0 days	0 days	0 days
¥The international definition of a DOT is any day on which one or more doses of antibiotic are administered					Total days of non-essential antibiotic therapy	Days*	Days*	Days*
**Total DOTs (sum of all antibiotics)**				days	**GRAND TOTAL non-essential antibiotic DOTs (sum of A + B + C)**	Days*		
**COURSE LENGTH (earliest start date to final stop date)**				days				

EXPLANATORY NOTES FOR DATA COLLECTORS

Survey of Appropriateness of Antibiotic Therapy for a Single Episode of infection in Adult Patients in an Acute Hospital Setting – DRAFT v8.1

Aim

This survey aims to estimate what proportion of antibiotic (antibacterial) days of therapy are non-essential in the judgement of the reviewer and therefore potentially avoidable.

Rationale
The focus on avoidable days of therapy is deliberate; to establish whether there is room for improvement in antibiotic consumption and to identify improvement goals that are safe for patients and relevant for antibiotic resistance. Assessment of appropriateness is subjective but this survey aims to standardise the assessment as much as possible by prompting the collection of relevant information to allow an infection specialist (doctor or pharmacist) to evaluate the appropriateness of antibiotic prescribing in a consistent way. *This survey instrument is not designed to capture information on off-guideline prescribing or overuse of broad-spectrum or IV antibiotics, nor is the survey designed to evaluate peri-operative surgical prophylaxis.*

Method
Selecting patients for the survey is critical. Please include adult patients at the end, or nearing the known end, of a course of treatment for a single episode of infection (completing treatment courses on the ward with a stop date or on discharge prescriptions). Seek the co-operation of ward pharmacists or dispensary staff (for discharge prescriptions) to identify patients at the end of a course of antibiotics, or use your e-prescribing system. Aim to sample randomly across a range of medical and surgical specialties. A sample of 50-100 patients per hospital is required to obtain a reliable estimate of the appropriateness of prescribing overall for your hospital.

Data for submission to survey organisers
Data items for submission are indicated with an asterisk. This is a minimum dataset to reduce workload associated with this survey. The majority of data items included in this survey instrument are for the benefit of the expert when estimating appropriateness and to allow the survey team to maintain a record of the source patient and date.

NEWS-2
For patients with no apparent evidence of local infection at an anatomical site, NEWS-2 is provided as an aid for identifying sepsis of uncertain origin and also to capture apparent discordance between clinical findings and prescribing behaviour. *This score has not been validated in paediatric patients.*

Applying NEWS ≥3 as a screening threshold for severe sepsis (Surviving Sepsis Campaign 2012 criteria) had sensitivity of 93% and a negative predictive value of 99.5% in an Emergency Department (ED) setting in London [Keep JW et al, 2016]. A study of over 27,000 adult patients admitted to 20 Scottish hospitals identified almost 20% meeting the Surviving Sepsis Campaign 2012 criteria for sepsis; only 9.7% of these patients had a NEWS <3 before leaving the ED [Corfield AR et al, 2014]. In December 2017, the Royal College of Physicians updated NEWS to NEWS-2 with a threshold of ≥5 for consideration of sepsis. [RCP 2017]

National Early Warning Score (NEWS2)

PHYSIOLOGICAL PARAMETERS	3	2	1	0	1	2	3
Respiration Rate (per minute)	≤8		9-11	12-20		21-24	≥25
SpO_2 Scale 1§ (%)	≤91	92-93	94-95	≥96			
SpO_2 Scale 2§ (%)	≤83	84-85	86-87	88-92 or ≥93 on air	93-94 on oxygen	95-96 on oxygen	≥97 on oxygen
Air or oxygen?		Oxygen		Air			
BP systolic (mmHg)	≤90	91-100	101-110	111-219			≥220
Pulse (per minute)	≤40		41-50	51-90	91-110	111-130	≥131
Consciousness (ACVPU)				A			C, V, P, or U
Temperature (°C)	≤35.0		35.1-36.0	36.1-38.0	38.1-39.0	≥39.1	
TOTAL SCORE							

§Use Scale 2 if target range is 88–92%, e.g. in hypercapnic (Type 2) respiratory failure (usually due to COPD).
ACVPU = Alert; new-onset Confusion, disorientation and/or agitation; responds to Voice; responds to Pain; Unresponsive

Royal College of Physicians. National Early Warning Score (NEWS) 2: Standardising the assessment of acute-illness severity in the NHS. London: RCP, 2017.
"A NEW score of 5 or more in patients with a known infection, signs or symptoms of infection, or at high risk of infection, is most likely to represent sepsis requiring a rapid escalation of clinical care, confirmatory investigations and urgent treatment." RCP 2017

SECTION II – ASSESSMENT OF APPROPRIATENESS OF *INTRAVENOUS ROUTE*			
**Exclusion criteria**		**IV-to-oral Switch Criteria**	**Date when criterion met**
Staph. aureus bacteraemia	Y / N	24 hours of IV therapy complete	
Meningitis or CNS infection	Y / N	Afebrile (<38˚C) for >24 hours	
Abscess or empyema (undrained)	Y / N	Clinical condition improving	
Haematological malignancy or neutropenia	Y / N	Haemodynamically stable	
Legionella pneumonia	Y / N	CRP trending towards normal	
Osteomyelitis or mediastinitis	Y / N	WBC trending towards normal	
Septic arthritis	Y / N	Able to tolerate oral medication	
Prosthetic device or foreign body infection	Y / N	Functioning GI tract without risk of malabsorption	
Necrotising fasciitis or other severe soft tissue infection	Y / N	**Date when ALL criteria met:**	
Endocarditis or intravascular infection	Y / N	Appropriate oral antibiotic available	Y / N
Cystic Fibrosis exacerbation	Y / N		
Haematological malignancy or neutropenia	Y / N		**Reference: Mertz D *et al*, 2009**

**DRUG CHART / PRESCRIPTION**						**Record any non-essential (excess) IV route days of therapy (DOTs) for one of 3 reasons**		
**Antibiotic**	**Intravenous antibiotic (IVAb) name (route & dose regimen)**	**Start date & time**	**Stop date & time**	**Days of therapy (DOTs)¥**	**Date & time IV-to-oral switch criteria met**<br><br>**(enter N/A if switch criteria not met)**	A.<br>IVAb(s) not indicated at outset (oral route appropriate)	B.<br>Unexplained continuation beyond switch date	C.<br>Unexplained continuation beyond standard course length for infection
**1**				days		days	days	days
**2**				days		days	days	days
**3**				days		days	days	days
**4**				days		days	days	days
**5**				days		days	days	days

¥The international definition of a DOT is any day on which one or more doses of antibiotic are administered	days	Total days of non-essential IV route antibiotic therapy	Days*	Days*	Days*
**Total DOTs (sum of all IV route antibiotics)**		**GRAND TOTAL non-essential IV antibiotic DOTs (sum of A + B + C)**	Days*		
