# Supplementary material for: Identification of Intervention Opportunities through Assessment of the Appropriateness of Antibiotic Prescribing in Surgical Patients in a UK Hospital Using a National Audit Tool: A Single Centre Retrospective Audit
Source: Antibiotics (Basel). 2022 Nov 8;11(11):1575. doi: 10.3390/antibiotics11111575 (PMC9686826; doi:10.3390/antibiotics11111575)
Supplement: Supplementary file 1 [file antibiotics-11-01575-s001.zip › Supplementary material file S1 - PHE Pilot Briefing Document-converted.pdf]

DEFINING AND MEASURING "APPROPRIATE" ANTIBIOTIC PRESCRIBING – A BRIEFING FOR AUDITORS

Dr Kieran Hand, Consultant Pharmacist – Anti-infectives, University Hospital Southampton NHS Foundation Trust. 26 September 2017.

Background

The UK government has pledged to reduce inappropriate antibiotic prescribing by 50% by the year 2020.[a]

Antimicrobial therapy and prophylaxis in hospitals has been reported to be incorrect or not indicated in 9-64% of cases and this variation is partly explained by differences in applied definitions of appropriateness as well as differences in subjective judgment of auditors.[1,2] Antibiotic prescribing in the absence of infection is likely to be less of a problem in secondary care compared to primary care but nonetheless has been reported to range from 9.3% in Wales (Wrexham) to 50% in Scotland (Aberdeen).[1,3-9] A recent six-hospital study from the US reported that at the time of starting antimicrobials, one third of patients did not have a fever or abnormal WBC count, and half of the requested radiology and microbiology results did not identify an infection.[9] This suggests that a large proportion of antibiotic use could have been avoided.

Variability in subjective judgment of appropriateness by infection experts is acknowledged in studies reporting inter-rater reliability.[10] ID physicians are reported to disagree on appropriate antibiotic therapy 30% of the time.[11]

The purpose of this document is to introduce an audit tool that has been drafted in an attempt to standardise the process of auditing appropriateness of antibiotic prescribing in NHS hospitals in order to quantify the degree of inappropriate prescribing and define the goal of reducing this by 50%.

Establishing prescribing standards & defining appropriateness

Defining the gold standard for appropriate antibiotic prescribing is a subjective process and a list was compiled to incorporate those elements of high-quality prescribing identified by opinion leaders in the field such as Dr Inge Gyssens,[12-14] government advisory committees such as APRHAI,[15,16] and panels of experts convened for the purpose of defining appropriate prescribing.[17] The table below (Table S1) sets out many of the elements of antibiotic prescribing that may be considered in an assessment of appropriateness. The elements are structured within the "Start Smart, Then Focus" format.[15]

It could reasonably be argued that each of these elements is relevant to improving patient outcomes and critical to the assessment of appropriateness of prescribing. However, APRHAI took a view that improvement in performance against certain elements would not impact upon the overall consumption of antibiotics and, consequently, would be less likely to impact upon antibiotic resistance. Three aspects were therefore given **ultimate priority** as most relevant to resistance and presented to APRHAI as follows:

"For the purpose of delivering the ambition of halving inappropriate prescribing in the UK, inappropriate prescribing is defined as;

---

[a] https://www.gov.uk/government/speeches/g7-2016-in-japan-pm-press-statement

- Prescribing an antibiotic for a patient in the absence of (documented) evidence of bacterial infection.
- Prescribing a critical broad-spectrum antibiotic (piperacillin-tazobactam or carbapenems in secondary care; co-amoxiclav, cephalosporins and quinolones in primary care) to patients in the absence of a (documented) rationale.
- Continuing an antibiotic prescription beyond the course length recommended in local or national guidelines, in the absence of a (documented) rationale."

A key function of the draft audit tool is to estimate the number of days of antibiotic therapy that auditors consider non-essential and therefore potentially avoidable. This will allow the NHS to set goals for reduction of antibiotic consumption in hospitals that are safe and achievable.

Table S1. Elements of antibiotic prescribing in hospitals relevant for evaluating appropriateness[12-17]

Prescribing elements (potential audit standards)	Comments	Selected for audit
**START SMART**		
No antibiotic if not indicated (no reasonable evidence of infection)	Unnecessary antibiotic exposure selects for avoidable resistance.[18-20]	
Indication documented	Good practice for continuity of care but of uncertain relevance to resistance.	
Appropriate specimens taken for MC&S (blood cultures and suspected site of infection)	Important for establishing evidence of infection and for targeting appropriate therapy but limited to manual audit and >50% negative.[9]	
No allergy or contra-indication to treatments	Important patient safety consideration but not relevant for resistance.	
Prompt administration of first dose	Important patient safety consideration in cases of severe sepsis but of uncertain relevance to resistance. Captured by sepsis CQUIN audits.	
Treatment regimen adequate to cover most likely pathogens	Meta-analysis of RCTs reports increased risk of mortality if initial regimen inadequate.[21] Relevance to resistance uncertain.	*
Treatment regimen not unnecessarily broad spectrum	Indiscriminate use of critical broad- spectrum agents unnecessarily selects for resistance.[22-24]	*
No redundant agents in treatment regimen	Unnecessary antibiotic exposure selects for avoidable resistance.[18-20]	
Treatment regimen compliant with local/national guideline or justified deviation	Validity dependent upon quality of local guideline. Relevance to resistance uncertain.	
Treatment regimen cost-effective	Not relevant to resistance.	
No underdosing	Limited evidence from modeling suggests that low doses may select resistance in pneumococci[25] but underdosing unlikely to be a problem in NHS hospitals due to pharmacist and nurse intervention.	
No overdosing	Important patient safety consideration but likely to reduce rather than increase risk of selecting resistance.[26-31]	
Correct route of administration	Relevant for efficacy, length of stay and risk of line infection but of uncertain relevance to resistance.	
Prompt appropriate source control	Subjective assessment. Of uncertain relevance to resistance.	

No missed doses or delayed doses	Of uncertain relevance to selection of resistance.	
Therapeutic drug monitoring (TDM) for narrow therapeutic index drugs	Important primarily for patient safety (but also for efficacy); of uncertain relevance to resistance.	
**THEN FOCUS**		
Prompt discontinuation of antibiotics if alternative diagnosis established and infection excluded	There is RCT evidence that unnecessary continuation selects for multi-resistant organisms.[32]	
Appropriate broadening of spectrum in response to MC&S results	This may necessitate an increase in broad-spectrum agent use if indicated by MC&S results. Failure to adjust ineffective treatment to MC&S results is associated with a higher risk of mortality.[33]	*
Appropriate narrowing of spectrum in response to MC&S results	Evidence largely from observational studies suggests that de-escalation to narrow- spectrum agents is safe when patients are improving clinically and a plausible pathogen has been identified.[34]	*
Prompt referral to OPAT services for suitable patients	Relevant for length of stay and risk of HCAI but of uncertain relevance to resistance.	
Prompt switch from IV to oral route of administration when safe and effective	Relevant for length of stay and risk of line infection but of uncertain relevance to resistance.	
Antibiotic plan documented in the notes	Good practice for continuity of care but of uncertain relevance to resistance.	
No unjustified prolonged duration of treatment	There is evidence from RCTs and observational studies that unnecessarily prolonged duration selects for multi- resistant organisms.[32,35,36] Can only be audited at the end of therapy.	
*Prescribing elements relating to antibiotic spectrum; removed for pilot study		

Selecting prescribing standards for audit

To reduce complexity for the purposes of a pilot evaluation of an appropriateness audit tool, the decision was taken to remove prescribing standards related to antibiotic spectrum (indicated by an asterisk in Table S1). A second component of this audit tool may subsequently be introduced to specifically address the evaluation of appropriateness antibiotic spectrum.

What are other countries doing?

The ECDC point prevalence survey (PPS) of HCAI and antimicrobial use has been criticised for failing to collect qualitative information about the appropriateness of the prescription.[37] The PPS does not capture information on the justification for starting or continuing an antibiotic for community- acquired infections and cannot provide accurate course length data due to auditing many patients mid-treatment.

Infection specialists in Australia have adapted the ECDC point prevalence survey tool to better measure prescribing quality and this tool has performed well in validation, inter-rater reliability and user feedback.[38] The Centers for Disease Control in Atlanta in the US have also published an audit

tool to evaluate the quality of inpatient antibiotic prescribing (http://www.cdc.gov/getsmart/healthcare/implementation.html .[2]

However, both the Australian and CDC tools have scope for refinement including assessment of evidence of infection, differentiating between evaluation of empirical or definitive pathogen- directed therapy and capturing justification for broad-spectrum prescribing. The existing tools also do not collect information on the potentially non-essential or avoidable days of antibiotic therapy and therefore cannot inform a goal for safe reduction of antibiotic consumption.

NEWS and qSOFA scores

In cases of undifferentiated sepsis, where signs or symptoms of infection do not appear to be localised to a discrete anatomical site, two patient acuity scoring systems have been incorporated into this draft audit tool to support the auditor in their assessment of evidence of infection and justification of antibiotic prescribing.

The National Early Warning Score (NEWS) was developed by the Royal College of Physicians and partners with the aim of standardising the assessment of acute-illness severity in the NHS.[39] A 2015 study of 15 Welsh hospitals reported that 26% (290/1111) of adult inpatients with a NEWS ≥3 had sepsis (including severe sepsis) defined as high clinical suspicion of infection with systemic inflammatory response syndrome (SIRS≥2).[40] This study, from Szakmany and colleagues, reported a median NEWS of 4 for patients meeting sepsis criteria compared with 5 for those meeting severe sepsis criteria.

Keep JW et al 2016 performed a retrospective study over one week in July 2013 of adult patients presenting to the ED of King's College Hospital, London, excluding patients with trauma.[41] Of 500 patients included, 101/500 (20%) were 'patients with infection', 50/101 (49.5%) were reported as 'septic' (SIRS≥2), and 27/50 (54%) had severe sepsis (according to Surviving Sepsis Campaign definition 2012), representing 10% of patients and 5.4% of included patients respectively. The study identified 134/500 patients (27%) with NEWS ≥3 and of these 25/134 (18.6%) had severe sepsis.
Applying NEWS ≥3 as a screening threshold for severe sepsis had a sensitivity of 92.6%, specificity of 77%, PPV of 18.7% and NPV of 99.5%. Two of 27 patients with severe sepsis had a NEWS of 2, representing a false negative rate in the target population of 7.5% for NEWS ≥3. Increasing the threshold to NEWS ≥4 resulted in an unacceptable false negative rate of 26% (7/27) of patients with severe sepsis.

Corfield AR et al 2014 screened 27,046 adult patients admitted to 20 Scottish hospitals for at least 2 days in 2009 for sepsis criteria using the Surviving Sepsis Campaign definition of 2012 and identified 5285 (19.5%) with SIRS≥2 (excluding patients with obvious non-infective pathology).[42] 9.7% of patients with sepsis had NEWS <3. Patients with sepsis had a median NEWS score of 7 and patients admitted to ICU had a significantly higher median NEWS score of 9 compared with non-ICU patients (median score 6). A single NEWS score of 7 or above in the ED for patients with sepsis was associated with a 27% chance of requiring ICU admission within 48 hours or 30-day mortality.

In summary, if a threshold of a NEWS score of 3 or above is applied for starting antibiotics, then it is anticipated that 7.5% of adult ED patients with severe sepsis will be denied antibiotics (Keep JW 2016) and 9.7% of adult ED patient with sepsis will be denied antibiotics (Corfield AR 2016). It therefore seems reasonable to assess what proportion of patients treated with antibiotics either have NEWS ≥3 or localised evidence of infection at an anatomical site. A prospective study to evaluate the use of NEWS to trigger antibiotic treatment has not been carried out so NEWS can only be used as a guide to the presence of a systemic inflammatory response.

The quick sequential organ failure assessment (qSOFA) score was proposed to the Third International Consensus Definitions for Sepsis and Septic Shock (Sepsis-3) as providing superior prediction of in-hospital mortality among adult patients with suspected infection outside of the ICU.[43] The predictive validity for in-hospital mortality of qSOFA was assessed in almost 75,000 adult patients hospitalised with suspected infection and found to be statistically greater than SOFA and SIRS, supporting its use as a prompt to consider possible sepsis.[44] A qSOFA score of 2 or more is proposed as the cut-off for sepsis (score one each for: RR >21; systolic BP 100 or lower; altered mental status).

A recent French study from Freund Y et al in 27 EDs (plus one each in Spain, Switzerland and Belgium) recruited consecutive patients presenting to ED with clinical suspicion of infection.[45] Evidence of infection was confirmed retrospectively by 2 experts and patients with localised infection and normal vital signs were excluded. Of the 879 patients included, 661 (75%) had qSOFA <2 and 22 (3.3%) of this cohort died (NPV 96.7%). 218 (25%) had qSOFA of 2 or more and 52 (24%) died. In contrast, 75% had SIRS of 2 or more. The AUROC was found to be significantly better for qSOFA (0.8) vs SIRS (0.65) to predict mortality, with similar results for predicting ICU admission.

Churpek MM et al 2017 screened adult admissions to a 500-bed university hospital in Chicago over an 8-year period and compared the performance of SIRS, qSOFA, MEWS and NEWS for predicting in- hospital mortality or ICU admission.[46] The final study cohort consisted of 30,677 patients who met the definition of suspicion of infection outside the ICU (ED or wards), with both antibiotics and cultures within a predefined time window. In-hospital mortality was 5% for the cohort and 24% were admitted to ICU. A NEWS score of 3 or above (87% of patients) had high sensitivity and identified 96.5% of patients who died; specificity was low at 6.2% and PPV was 5.5% (Appendix Table 1) so 94.5% of patients with a NEWS of 3 or more survived. The NPV of NEWS ≥3 was 96.9% in this cohort. A qSOFA score of 2 or above (38% of patients) had lower sensitivity and only identified 68.7% of patients who died; specificity was also low at 12.3% and PPV was 9.7% (Appendix Table 1) so 90.3% of patients with a qSOFA of 2 or more survived. NPV of qSOFA ≥2 was 97.3% in this cohort. The AUROC was found to be significantly better for NEWS (0.77) vs qSOFA (0.69) to predict mortality.

In summary, applying qSOFA score ≥2 during ED or ward stay as a prediction tool for in-hospital all- cause mortality had a negative predictive value of 97% in adult patients with clinical suspicion of infection. A qSOFA score at a threshold of 2 or more, may be considered by some clinicians to be a reasonable tool for differentiating patients in whom immediate broad-spectrum antibiotics are justified, given the associated in-hospital mortality of 24% reported in the Freund ED study in France. However, as with NEWS, qSOFA has not been tested in a prospective study where it is used to dictate antibiotic treatment. The qSOFA is not an alert that alone will differentiate patients with infection from those without infection.

Limitations of the proposed audit

- The draft audit tool is intended to be used for adult patients in the first instance (qSOFA and NEWS not validated predictors of sepsis mortality in children).
- The draft audit tool considers antibiotics used for treatment of infection and is not designed for auditing peri-operative surgical prophylaxis.
- The audit must be carried out at the end of a course of treatment (or when a prescription stop date has been documented) in order to evaluate course length and this presents challenges with identifying patients prior to hospital discharge.

- Assessment of appropriateness is left to the discretion of the auditor and it is strongly recommended that the audit is undertaken by a health professional with expertise in infection. The assessment process should be aided by the available evidence of infection or sepsis, including qSOFA score, NEWS, CRP and white blood cell count. However, the decision remains a subjective one and the validity of this audit relies on the integrity of the auditor.

Validation of the proposed audit tool

The propsed audit tool will be subject to assessment of face-validity with a panel of UK infection specialists and in-use validation by comparing the appropriateness assessments of pairs of infection specialists (e.g. pharmacists and microbiologists or infectious diseases physicians).

1. Cusini A, Rampini SK, Bansal V, et al. Different patterns of inappropriate antimicrobial use in surgical and medical units at a tertiary care hospital in Switzerland: a prevalence survey. *PLoS. One* 2010;5(11):e14011.
2. Spivak ES, Cosgrove SE, Srinivasan A. Measuring Appropriate Antimicrobial Use: Attempts at Opening the Black Box. *Clin Infect Dis* 2016;63(12):1639-44.
3. Akhloufi H, Streefkerk RH, Melles DC, et al. Point prevalence of appropriate antimicrobial therapy in a Dutch university hospital. *Eur. J. Clin. Microbiol. Infect. Dis* 2015;34(8):1631-37.
4. Hecker MT, Aron DC, Patel NP, et al. Unnecessary use of antimicrobials in hospitalized patients: current patterns of misuse with an emphasis on the antianaerobic spectrum of activity. *Arch. Intern. Med* 2003;163(8):972-78.
5. Kumarasamy Y, Cadwgan T, Gillanders IA, et al. Optimizing antibiotic therapy-the Aberdeen experience. *Clin. Microbiol. Infect* 2003;9(5):406-11.
6. Lesprit P, de PA, Esposito-Farese M, et al. Postprescription review improves in-hospital antibiotic use: a multicenter randomized controlled trial. *Clin. Microbiol. Infect* 2015;21(2):180-87.
7. Roberts E, Dawoud DM, Hughes DA, et al. Evaluation of a consultant audit and feedback programme to improve the quality of antimicrobial prescribing in acute medical admissions. *Int J Pharm Pract* 2015;23(5):333-9.
8. Vlahovic-Palcevski V, Francetic I, Palcevski G, et al. Antimicrobial use at a university hospital: appropriate or misused? A qualitative study. *Int J. Clin. Pharmacol. Ther* 2007;45(3):169-74.
9. Braykov NP, Morgan DJ, Schweizer ML, et al. Assessment of empirical antibiotic therapy optimisation in six hospitals: an observational cohort study. *Lancet Infect. Dis* 2014;14(12):1220-27.
10. Mol PG, Gans RO, Panday PV, et al. Reliability of assessment of adherence to an antimicrobial treatment guideline. *J. Hosp. Infect* 2005;60(4):321-28.
11. Casaroto E, Marra AR, Camargo TZ, et al. Agreement on the prescription of antimicrobial drugs. *BMC Infect Dis* 2015;15:248.
12. Gyssens IC. Quality measures of antimicrobial drug use. *Int. J. Antimicrob. Agents* 2001;17(1):9- 19.
13. Kunin CM, Tupasi T, Craig WA. Use of antibiotics. A brief exposition of the problem and some tentative solutions. *Ann. Intern. Med* 1973;79(4):555-60.
14. Willemsen I, Groenhuijzen A, Bogaers D, et al. Appropriateness of antimicrobial therapy measured by repeated prevalence surveys. *Antimicrob. Agents Chemother* 2007;51(3):864- 67.
15. Ashiru-Oredope D, Sharland M, Charani E, et al. Improving the quality of antibiotic prescribing in the NHS by developing a new Antimicrobial Stewardship Programme: Start Smart--Then Focus. *J. Antimicrob. Chemother* 2012;67 Suppl 1:i51-i63.
16. Public_Health_England. Antimicrobial Stewardship: "Start Smart - Then Focus". *Advisory Committee on Antimicrobial Resistance and Healthcare Associated Infection (ARHAI)* 2015. https://www.gov.uk/government/publications/antimicrobial-stewardship-start-smart-then- focus.
17. van den Bosch CM, Geerlings SE, Natsch S, et al. Quality indicators to measure appropriate antibiotic use in hospitalized adults. *Clin Infect Dis* 2015;60(2):281-91.
18. Lopez-Lozano JM, Monnet DL, Yague A, et al. Modelling and forecasting antimicrobial resistance and its dynamic relationship to antimicrobial use: a time series analysis. *Int. J. Antimicrob. Agents* 2000;14(1):21-31.
19. Tacconelli E, Cataldo MA, De PG, et al. Prediction models to identify hospitalized patients at risk of being colonized or infected with multidrug-resistant Acinetobacter baumannii calcoaceticus complex. *J. Antimicrob. Chemother* 2008;62(5):1130-37.
20. Tacconelli E, De AG, Cataldo MA, et al. Antibiotic usage and risk of colonization and infection with antibiotic-resistant bacteria: a hospital population-based study. *Antimicrob. Agents Chemother* 2009;53(10):4264-69.
21. Paul M, Shani V, Muchtar E, et al. Systematic review and meta-analysis of the efficacy of appropriate empiric antibiotic therapy for sepsis. *Antimicrob. Agents Chemother* 2010;54(11):4851-63.
22. Harris AD, Perencevich E, Roghmann MC, et al. Risk factors for piperacillin-tazobactam-resistant Pseudomonas aeruginosa among hospitalized patients. *Antimicrob. Agents Chemother* 2002;46(3):854-58.

23. Lai CC, Wang CY, Chu CC, et al. Correlation between antibiotic consumption and resistance of Gram-negative bacteria causing healthcare-associated infections at a university hospital in Taiwan from 2000 to 2009. *J. Antimicrob. Chemother* 2011;66(6):1374-82.
24. Pakyz AL, Oinonen M, Polk RE. Relationship of carbapenem restriction in 22 university teaching hospitals to carbapenem use and carbapenem-resistant Pseudomonas aeruginosa. *Antimicrob. Agents Chemother* 2009;53(5):1983-86.
25. Opatowski L, Mandel J, Varon E, et al. Antibiotic dose impact on resistance selection in the community: a mathematical model of beta-lactams and Streptococcus pneumoniae dynamics. *Antimicrob. Agents Chemother* 2010;54(6):2330-37.
26. Guillemot D, Carbon C, Balkau B, et al. Low dosage and long treatment duration of beta-lactam: risk factors for carriage of penicillin-resistant Streptococcus pneumoniae. *JAMA* 1998;279(5):365-70.
27. Handel A, Margolis E, Levin BR. Exploring the role of the immune response in preventing antibiotic resistance. *J. Theor. Biol* 2009;256(4):655-62.
28. Martinez MN, Papich MG, Drusano GL. Dosing regimen matters: the importance of early intervention and rapid attainment of the pharmacokinetic/pharmacodynamic target. *Antimicrob. Agents Chemother* 2012;56(6):2795-805.
29. Olofsson SK, Cars O. Optimizing drug exposure to minimize selection of antibiotic resistance. *Clin. Infect. Dis* 2007;45 Suppl 2:S129-S36.
30. Schrag SJ, Pena C, Fernandez J, et al. Effect of short-course, high-dose amoxicillin therapy on resistant pneumococcal carriage: a randomized trial. *JAMA* 2001;286(1):49-56.
31. Tam VH, Louie A, Deziel MR, et al. The relationship between quinolone exposures and resistance amplification is characterized by an inverted U: a new paradigm for optimizing pharmacodynamics to counterselect resistance. *Antimicrob. Agents Chemother* 2007;51(2):744-47.
32. Singh N, Rogers P, Atwood CW, et al. Short-course empiric antibiotic therapy for patients with pulmonary infiltrates in the intensive care unit. A proposed solution for indiscriminate antibiotic prescription. *Am. J. Respir. Crit Care Med* 2000;162(2 Pt 1):505-11.
33. Palacios-Baena ZR, Gutierrez-Gutierrez B, De Cueto M, et al. Development and validation of the INCREMENT-ESBL predictive score for mortality in patients with bloodstream infections due to extended-spectrum-beta-lactamase-producing Enterobacteriaceae. *J Antimicrob Chemother* 2017;72(3):906-13.
34. Schuts EC, Hulscher ME, Mouton JW, et al. Current evidence on hospital antimicrobial stewardship objectives: a systematic review and meta-analysis. *Lancet Infect. Dis* 2016;16(7):847-56.
35. Chastre J, Wolff M, Fagon JY, et al. Comparison of 8 vs 15 days of antibiotic therapy for ventilator-associated pneumonia in adults: a randomized trial. *JAMA* 2003;290(19):2588-98.
36. Marra AR, de Almeida SM, Correa L, et al. The effect of limiting antimicrobial therapy duration on antimicrobial resistance in the critical care setting. *Am J Infect Control* 2009;37(3):204-9.
37. Kieran JA, O'Doherty RG, Hudson BJ. ESAC point prevalence methodology to assess antimicrobial consumption and quality of prescribing in an Australian setting. *Med. J. Aust* 2011;194(2):103-04.
38. James R, Upjohn L, Cotta M, et al. Measuring antimicrobial prescribing quality in Australian hospitals: development and evaluation of a national antimicrobial prescribing survey tool. *J. Antimicrob. Chemother* 2015;70(6):1912-18.
39. Physicians RCo. National Early Warning Score (NEWS): Standardising the assessment of acute illness severity in the NHS. Report of a working party. https://www.rcplondon.ac.uk/projects/outputs/national-early-warning-score-news, 2012.
40. Szakmany T, Lundin RM, Sharif B, et al. Sepsis Prevalence and Outcome on the General Wards and Emergency Departments in Wales: Results of a Multi-Centre, Observational, Point Prevalence Study. *PLoS One* 2016;11(12):e0167230.
41. Keep JW, Messmer AS, Sladden R, et al. National early warning score at Emergency Department triage may allow earlier identification of patients with severe sepsis and septic shock: a retrospective observational study. *Emerg Med J* 2016;33(1):37-41.
42. Corfield AR, Lees F, Zealley I, et al. Utility of a single early warning score in patients with sepsis in the emergency department. *Emerg Med J* 2014;31(6):482-7.
43. Singer M, Deutschman CS, Seymour CW, et al. The Third International Consensus Definitions for Sepsis and Septic Shock (Sepsis-3). *JAMA* 2016;315(8):801-10.
44. Seymour CW, Liu VX, Iwashyna TJ, et al. Assessment of Clinical Criteria for Sepsis: For the Third International Consensus Definitions for Sepsis and Septic Shock (Sepsis-3). *JAMA* 2016;315(8):762-74.
45. Freund Y, Lemachatti N, Krastinova E, et al. Prognostic Accuracy of Sepsis-3 Criteria for In- Hospital Mortality Among Patients With Suspected Infection Presenting to the Emergency Department. *JAMA* 2017;317(3):301-08.
46. Churpek MM, Snyder A, Han X, et al. Quick Sepsis-related Organ Failure Assessment, Systemic Inflammatory Response Syndrome, and Early Warning Scores for Detecting Clinical Deterioration in Infected Patients outside the Intensive Care Unit. *Am J Respir Crit Care Med* 2017;195(7):906-11.
